# Clinical, Dietary, Lifestyle and Genetic Factors Associated With Age at Onset of Esophageal Adenocarcinoma

**DOI:** 10.1002/ueg2.70236

**Published:** 2026-06-11

**Authors:** Vera Koch, Ines Gockel, Julian Reingruber, Nicole Kreuser, Jessica Bigge, Marino Venerito, Hakan Alakus, Andrea May, Christian Gerges, René Thieme, Dominik Heider, Axel M. Hillmer, Alanna Ebigbo, Christiane J. Bruns, Hauke Lang, Helmut Messmann, Thomas Rösch, Thaddäus T. Wissniowski, Michaela Müller, Ulrike W. Denzer, Markus M. Nöthen, Michael Vieth, Aaron P. Thrift, Carlo Maj, Johannes Schumacher

**Affiliations:** ^1^ Center for Human Genetics Philipps University of Marburg & University Hospital Marburg Marburg Germany; ^2^ Department of Visceral Surgery University Digestive Health Care Center Basel Clarunis Switzerland; ^3^ Department of Visceral Transplant Thoracic and Vascular Surgery University Hospital Leipzig Leipzig Germany; ^4^ Department of Gastroenterology Hepatology and Infectious Diseases Otto‐von‐Guericke University Hospital Magdeburg Germany; ^5^ Department of General Visceral Cancer and Transplantation Surgery University Hospital of Cologne Cologne Germany; ^6^ Department of Gastroenterology Oncology and Pneumology Asklepios Paulinen Klinik Wiesbaden Wiesbaden Germany; ^7^ Department of Medicine II Helios Klinikum Krefeld Krefeld Germany; ^8^ Institute of Medical Informatics University of Münster Münster Germany; ^9^ Faculty of Medicine and University Hospital Cologne Institute of Pathology University of Cologne Cologne Germany; ^10^ Department of Gastroenterology University Hospital of Augsburg Augsburg Germany; ^11^ Department of Internal Medicine St. Josef‐Hospital Ruhr‐University Bochum Bochum Germany; ^12^ Department for General Visceral and Tranplant Surgery University Medical Center of the Johannes Gutenberg‐University Mainz Mainz Germany; ^13^ Department of Interdisciplinary Endoscopy University Hospital Hamburg‐Eppendorf Hamburg Germany; ^14^ Center for Internal Medicine II Klinikum Chemnitz Chemnitz Germany; ^15^ Division of Gastroenterology and Endocrinology Centre for Internal Medicine Philipps University of Marburg & University Hospital Marburg Marburg Germany; ^16^ Institute of Human Genetics University of Bonn School of Medicine & University Hospital Bonn Bonn Germany; ^17^ Institute of Pathology Friedrich‐Alexander‐Universiät Erlangen‐Nürnberg Klinikum Bayreuth Bayreuth Germany; ^18^ Section of Epidemiology and Population Sciences Department of Medicine Baylor College of Medicine Houston Texas USA; ^19^ Dan L Duncan Comprehensive Cancer Center Baylor College of Medicine Houston Texas USA

**Keywords:** age at onset, chemoprevention, esophageal adenocarcinoma, lifestyle, polygenic risk score

## Abstract

**Background:**

Esophageal adenocarcinoma (EAC) represents one of the most increasing malignancies in Western countries. The disease is multifactorial, involving modifiable risk factors and genetic susceptibility variants. These variants can be aggregated to a polygenic risk score (PRS) that reflects individual genetic risk. Investigation of the effects of lifestyle factors, PRS, and co‐medication on EAC age at onset (AAO) is critical for shaping prevention strategies.

**Methods:**

A detailed questionnaire was used to assess pre‐diagnostic exposure to lifestyle factors and clinical information from a large German EAC cohort. Linear regression analysis was performed to identify factors associated with EAC AAO in 1742 EAC patients. PRS was available for 1190 patients. Subgroup analyses were conducted to estimate the effects of the analyzed factors on AAO according to age group (early vs. late onset), sex, and prior diagnosis of Barrett's esophagus (BE).

**Results:**

Earlier AAO was significantly associated with gastroesophageal reflux (GER), smoking and a higher PRS, whereas later AAO was associated with physical activity and higher consumption of fish and fruits. Among co‐medication, combined use of proton pump inhibitors (PPIs) and acetylsalicylic acid (ASA) showed the most significant effect on AAO, whereas the use of PPIs and ASA alone showed weaker effects.

**Discussion:**

This study represents the largest questionnaire‐based analysis to date investigating factors influencing EAC development. Our findings show that the combined use of PPIs and ASA, both cost‐effective medications, is associated with delayed EAC onset. In addition, lifestyle and genetics contribute to EAC AAO.

## Key Summary

- Summarize the established knowledge on this subject.
  ◦ Esophageal adenocarcinoma (EAC) represents one of the most increasing malignancies in Western countries.
  ◦ The disease is multifactorial, involving modifiable risk factors and genetic susceptibility variants.
  ◦ Co‐medication with proton pump inhibitors (PPIs) combined with acetylsalicylic acid (ASA) has a beneficial effect on disease risk and all‐cause mortality.
  ◦ The identification of genetic risk variants has enabled the development of polygenic risk scores (PRS) that quantify the cumulative genetic burden and hold promise for improving the identification and stratification of individuals at high risk.
- What are the significant and/or new findings of this study?
  ◦ Significant effects on earlier age at onset (AAO) among EAC patients were observed for gastroesophageal reflux (GER), smoking and a higher PRS, while effects on later AAO were significant for physical activity, fish and fruit consumption.
  ◦ Among chemopreventive drugs, combined usage of PPIs and acetylsalicylic acid ASA showed the most significant effect on later AAO, whereas PPIs and ASA intake alone have less significant effects, respectively.
  ◦ We found a significant association between a higher PRS and earlier AAO of EAC, which was particularly pronounced among younger patients (AAO < 50 years).

## Graphical abstract

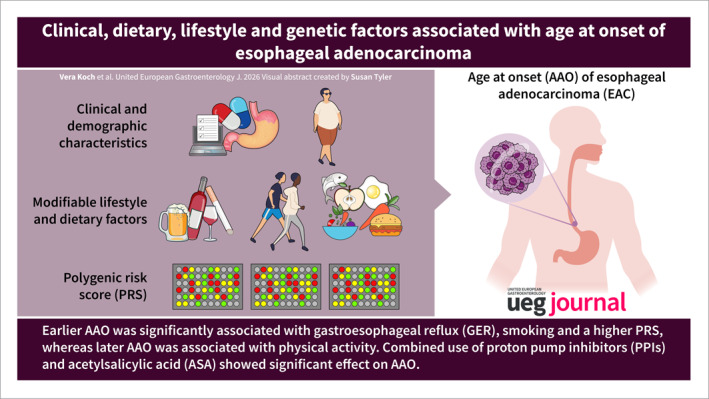

## 1 Introduction

Esophageal cancer (EC) is the 11th most newly diagnosed cancer worldwide and the seventh most common cause of cancer‐related death. The incidence rate is almost three times higher in men than in women [1]. Two histological subgroups are mainly present: esophageal squamous cell carcinoma (ESCC) and esophageal adenocarcinoma (EAC). Although ESCC is the most common EC type worldwide, EAC represents one of the most increasing malignancies in Western countries, paralleling changes in obesity, gastroesophageal reflux (GER), and Western diet [2]. EAC develops from its precursor lesion Barrett's esophagus (BE) [2]. However, BE is usually clinically asymptomatic and thus remains undiagnosed in the majority of EAC cases. Accordingly, malignant transformation is not recognized early in many patients, precluding endoscopic ablation as a therapeutic option.

One clinical factor that may reduce the risk of developing EAC is the use of chemopreventive agents to suppress acid and chronic inflammation in esophageal mucosa [3]. In 2018, a randomized trial by Jankowski et al. showed a beneficial effect of high‐dose therapy with proton pump inhibitors (PPIs) on disease risk and all‐cause mortality. This protective effect was further increased by the additional intake of aspirin [4].

In addition to external risk factors, genetic susceptibility contributes to EAC development. The disease is multifactorial and arises from the cumulative impact of numerous genetic risk variants [5]. The largest genome‐wide association study (GWAS) to date identified 23 such variants associated with EAC and BE [6]. For many multifactorial diseases, the identification of genetic risk variants has enabled the development of polygenic risk scores (PRS) that quantify the cumulative genetic burden and hold promise for improving the identification and stratification of individuals at high risk.

Given its high mortality at advanced stages [2], EAC has profound implications for public health. Understanding its rising burden and the interaction between modifiable lifestyle factors and genetic susceptibility is therefore crucial for shaping prevention strategies.

In the present study, we assessed the impact of genetics, clinical factors and pre‐diagnostic lifestyle exposures as risk factors for EAC. In addition, we assessed the role of PPIs and acetylsalicylic acid (ASA) as a non‐steroidal anti‐inflammatory drug (NSAID), which have previously been reported to exert protective effects [1, 2, 7]. The study sample comprised 1742 uniformly phenotyped EAC patients of European descent (1539 males, 203 females). The primary endpoint was age at onset (AAO) of EAC, which was tested for association with all analyzed exposures and served as a surrogate for disease risk. Using AAO as the primary endpoint leverages time‐to‐event information to increase statistical power relative to binary case‐control analyses and reduces biases from selecting and misclassifying controls [8].

## 2 Materials

All patients were of Central European descent and resided in Germany. A detailed questionnaire was used to record pre‐diagnosis exposure to lifestyle factors and clinical information from two independent cohorts (BonnV1, BonnV2) at the time of recruitment. In total, 1742 records from 1778 EAC patients were selected after excluding records with incomplete or duplicated data. For the remaining records, no imputation was used to account for incomplete data. Of all included patients, 1539 were attributable to males (88.35%) and 203 to females (11.65%).

The data were collected in a standardized procedure through patients' self‐reports with a detailed questionnaire (Supporting Information S1). For nutrition and lifestyle variables, frequencies of consumption were recorded as follows: maximum 1×/week, approximatly 3× per week, approx. 5× per week, daily for red meat; maximum 1× per week, 1–3× per week, every day for fruit, vegetables, and fish consumption. For smoking, including cigarettes, chewing tobacco, or pipe smoking, a distinction between former and current smokers was made. Lifelong pack‐years (PY) dosage was calculated as *n* cigarettes smoked per day/20 * *n* years of smoking, if data were available. Data from self‐reports were dichotomized for nutrition and lifestyle factors. Here, regular medication intake was defined as at least once per week. Additionally, we distinguish between ASA intake alone, PPIs intake alone, as well as combined intake of ASA and PPIs. Consumption of alcohol at least once per week was defined as regular. Consumption of red meat, fruits, and vegetables more than three times per week was classified as frequent intake, whereas fish consumption more than one time per week was considered frequent. A Western diet was defined as meat consumption at least three times per week. Physical activity performed at least once per week was classified as regular. Smoking was defined as any lifetime tobacco use. Educational attainment was categorized as low (completion of primary education) or high (secondary or higher education).

For BE diagnosis, C and M characteristics according to the Prague classification [9] were not available from patient self‐reports. We used self‐reported heartburn (less than once a week, once a week, 2–3× per week, 4–6× per week) as a proxy for GER, where heartburn more than once a week was considered as frequent for dichotomization.

PRS was available for 1190 patients. This score was previously developed in our earlier study, based on 23 single nucleotide polymorphisms (SNPs) identified through GWAS [6]. All included SNPs are available in (Supporting Information S1: Table S1). For details on SNP selection and PRS construction, please refer to the original publication.

## 3 Methods

The statistical analysis was conducted in R (v4.5.0) and aimed to assess the influence of clinical, demographical, lifestyle and dietary factors, as well as medication and PRS on AAO of EAC. Means and standard deviations were calculated for continuous variables, while frequencies and percentages were presented for categorical variables. PRS was standardized by calculation of a z‐score (*Z* = (*x* − *μ*)/*σ*), where × represents the individual PRS, *μ* the mean PRS of the analyzed cohort, and *σ* the standard deviation of the analyzed cohort.

A correlation matrix was generated for variables of interest (*N* = 19), where the Pearson correlation coefficient (*r*) was calculated (corrr v0.4.4). *r* < |0.4| was considered as a weak correlation, whereas *r* > |0.6| indicates a strong correlation between two variables.

For comparison between AAO means for the two groups, we used a two‐sided *t*‐test (t.test() from the stats package) with 95% confidence level for *p*‐value calculation. Multiple linear regression models were employed (package base) to identify significant influencing factors with correction to (i) age at examination (AAE), (ii) biological sex, and (iii) data collection cohort. Statistical significance was determined at P_adj_‐value *p* < 0.05. To account for multiple testing across all variables, we applied the Benjamini–Hochberg procedure to control the false discovery rate (FDR). The effects were visualized as forest plots (ggplot2 v3.5.2).

For subgroup analysis, we estimated the effects of analyzed factors on AAO for younger (AAO < 50 years, *N* = 190) and older EAC patients (AAO ≥ 50 years, *N* = 1552). Moreover, we investigated the effects on AAO for patients with prior BE diagnosis (*N* = 432) and without (*N* = 1310). To calculate the effect of BE on AAO, we included BE as analyzed variable in the linear regression model for the whole cohort (*N* = 1742). For sex‐specific analysis, we estimated the effects of analyzed factors on AAO for males (*N* = 1539) and females (*N* = 203) separately. Here, a linear model with correction only to (i) AAE and (ii) data collection cohort was applied.

To investigate the potential relationship between ASA intake and cardiovascular comorbidities, we calculated the odds ratios (OR) from 2 × 2 tables with package (epitools v0.5–10.1). The table contained the number of patients without cardiovascular comorbidities, who did not take ASA on a regular basis (*N* = 1376), patients without cardiovascular comorbidities, who took ASA on a regular basis (*N* = 176), patients with cardiovascular comorbidities, who did not take ASA on a regular basis (*N* = 102), and patients with cardiovascular comorbidities, who did take ASA regularly (*N* = 88). Two‐sided *p*‐value was calculated with Fisher conditional maximum likelihood estimation. Confidence intervals were calculated using Fisher’s exact method.

## 4 Results

**FIGURE 1:**
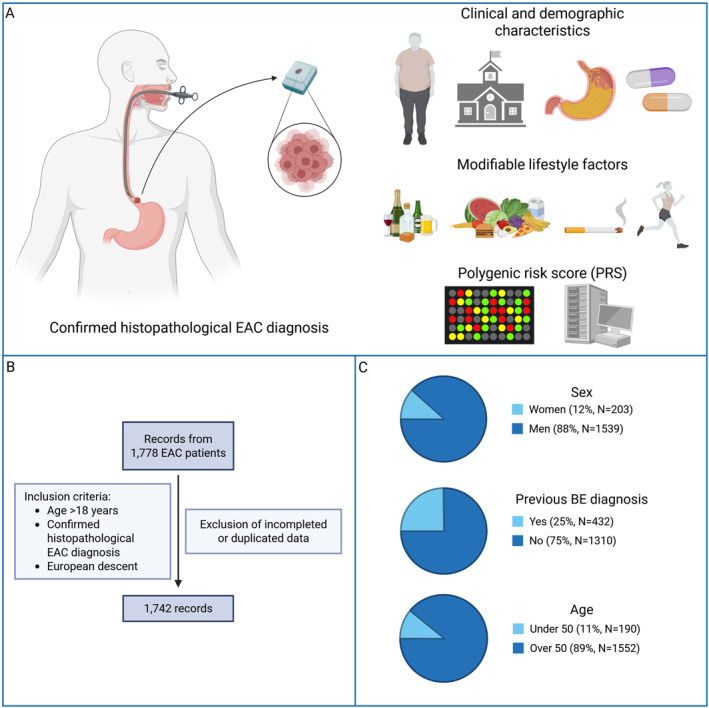
Data composition, inclusion and exclusion criteria for this study and cohort characterization. (A) Data utilized in this study include confirmed histopathological EAC diagnosis, demographic and clinical data from medical reports, self‐reported pre‐diagnosis exposures to potential risk factors from detailed questionnaires and interviews with trained medical specialists. PRS was calculated based on 23 SNPs associated with EAC and BE. (B) Flow diagram for inclusion and exclusion criteria in the resulting EAC patient cohort. (C) Pie plots display the proportion and number of male and female patients, patients with and without confirmed BE diagnosis, and patients with early AAO (< 50 years) and late AAO (≥ 50 years). AAO, age at onset; ASA, acetylsalicylic acid; BE, Barrett's esophagus; BMI, body mass index; EAC, esophageal adenocarcinoma; GER, gastroesophageal reflux; GWAS, genome‐wide association study; PPIs, proton pump inhibitors; PRS, polygenic risk score; SNPs, single nucleotide polymorphisms. This picture was created in BioRender. K.V. (2026) https://BioRender.com/l5984ih.

This study analyzed the impact of modifiable risk factors, medication, and genetic risk on EAC AAO. Figure 1 lists data utilized in this study, inclusion and exclusion criteria, as well as cohort characteristics of 1742 EAC patients. The mean AAO of our EAC cases was 62.14 (± 9.83) years, and the mean AAE was 65.75 (± 9.83) years.

Overall, we evaluated the impact of 13 exposures on EAC AAO, comprising clinical characteristics (body mass index (BMI), GER), co‐medication (PPI and/or ASA intake), educational level, lifestyle factors (smoking, alcohol consumption, physical activity), and dietary factors (intake of meat, fish, vegetables, and fruits). All records were obtained through patients' self‐reports with a detailed questionnaire (Supporting Information S1) and structured interviews conducted by trained medical professionals. In addition, we assessed the genetic burden of 1190 patients by applying a PRS comprising the 23 previously identified EAC/BE GWAS risk variants [6].

The correlation matrix of all included exposures is displayed in Supporting Information S1: Figure S1. Age‐dependent variables AAO and AAE are highly correlated (*r* = 0.919). Variables measuring medication intake, “PPIs intake” and “PPIs without ASA” (*r* = 0.858) as well as “ASA intake” and “ASA without PPIs” (*r* = 0.725) also showed a high correlation, since the variable composition was influenced by the same input parameters. Only a low correlation between all lifestyle variables was observed (Supporting Information S1: Figure S1).

**FIGURE 2:**
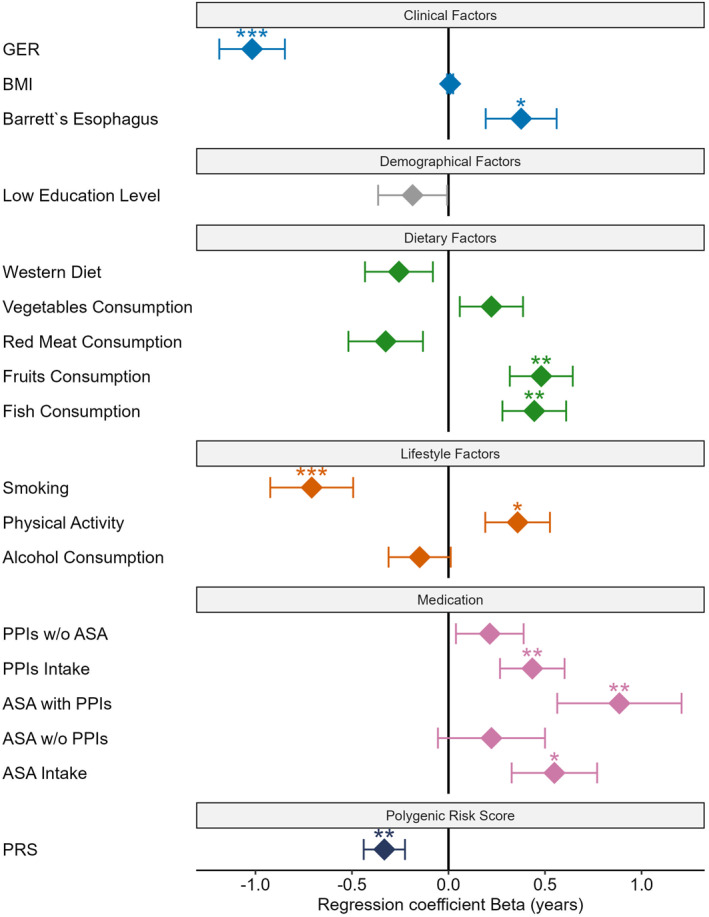
Estimated effects (*β*) of clinical, demographic, lifestyle, and dietary factors as well as PRS on AAO of EAC. Each point represents the *β* coefficient from linear regression with 95% confidence interval (CI). A negative *β* indicates earlier AAO, whereas a positive *β* is associated with later AAO. Significance levels: * (*p* < 0.05), ** (*p* < 0.01), and *** (*p* < 0.001). AAO, age at onset; ASA, acetylsalicylic acid; BMI, body mass index; EAC, esophageal adenocarcinoma; GER, gastroesophageal reflux; PPIs: proton pump inhibitors; PRS, polygenic risk score.

Table 1 represents the results of linear regression for EAC AAO. Among the established EAC risk factors [1], GER (*β* = −1.02 (± 0.17), *p* = 2.62 × 10^−9^) and smoking (*β* = −0.71 (± 0.21), *p* = 9.89 × 10^−4^) showed the strongest associations with an earlier AAO (Figure 2). In contrast, no significant association was observed for BMI (*β* = 0.01 (± 0.02), *p* = 0.587). Our data confirm that physical activity protects against EAC, as it was significantly associated with a later EAC AAO (*β* = 0.36 (± 0.17), *p* = 0.033) (Figure 2). In contrast, alcohol consumption showed no AAO association in our patient cohort. Of the nutrition factors, diets rich in fruit (*β* = 0.48 (± 0.16), *p* = 3.26 × 10^−3^) and fish (*β* = 0.44 (± 0.16), *p* = 7.13 × 10^−3^) showed significant association with later EAC AAO (Figure 2). Also, the vegetable‐rich diet showed association with later EAC AAO, although this was not significant. Overall, our data point to an association of the Mediterranean diet with later EAC AAO.

**TABLE 1:** Results of linear regression on AAO for 1742 included records from EAC patients.

Variable	Beta	SE	*p*	Group
GER	−1.017	0.170	2.62 × 10^−9^ ***	Clinical
Tobacco usage	−0.709	0.215	9.89 × 10^−4^ ***	Lifestyle
PRS	−0.332	0.107	1.93 × 10^−3^ **	Polygenic risk score
Red meat consumption	−0.325	0.193	9.2 × 10^−2^	Nutrition
Western diet	−0.257	0.176	1.44 × 10^−1^	Nutrition
Low education level	−0.186	0.179	2.98 × 10^−1^	Demographic
Alcohol consumption	−0.149	0.161	3.54 × 10^−1^	Lifestyle
BMI	0.009	0.016	5.87 × 10^−1^	Clinical
PPIs w/o ASA	0.214	0.175	2.22 × 10^−1^	Clinical
Vegetables consumption	0.222	0.164	1.75 × 10^−1^	Nutrition
ASA w/o PPIs	0.223	0.277	4.22 × 10^−1^	Clinical
Physical activity	0.358	0.168	3.27 × 10^−2^ *	Lifestyle
PPIs intake	0.434	0.167	9.51 × 10^−3^ **	Clinical
Fish consumption	0.445	0.165	7.13 × 10^−3^ **	Nutrition
Fruits consumption	0.481	0.163	3.26 × 10^−3^ **	Nutrition
ASA intake	0.549	0.221	1.33 × 10^−2^ *	Clinical
ASA with PPIs	0.885	0.322	6.03 × 10^−3^ **	Clinical

*Note:* Variables are sorted in ascending order by beta value.

Abbreviations: AAO, age at onset; ASA, acetylsalicylic acid; Beta, *β*‐coefficient; BMI, body mass index; GER, gastroesophageal reflux; *p,* false discovery rate (FDR) corrected *p*‐values, group, category of analyzed lifestyle factors or clinical information; PPIs, proton pump inhibitors, PRS, Polygenic Risk Score; SE, standard error.

Significance levels were defined as follows * (*p* < 0.05), ** (*p* < 0.01), and *** (*p* < 0.001).

We then analyzed the effects of medication intake on AAO. The combined intake of PPIs and ASA showed the strongest association with later EAC AAO (*β* = 0.89 (± 0.32), *p* = 6.03 × 10^−3^) (Figure 2). In contrast, the isolated use of PPIs or ASA showed no significant associations and their effect estimates were lower compared to the combined intake (Figure 2). The ASA use in our EAC patients was not due to a pre‐existing BE diagnosis, but rather to comorbid cardiovascular diseases (odds ratio (OR) = 6.73, *p* = 2.35 × 10^−28^, see Methods).

We next assessed the contribution of genetic risk factors to the AAO of EAC. Along with GER and smoking, the PRS was significantly associated with an earlier EAC AAO (*β* = −0.33 (± 0.11), *p* = 1.93 × 10^−3^) (Figure 2).

**FIGURE 3:**
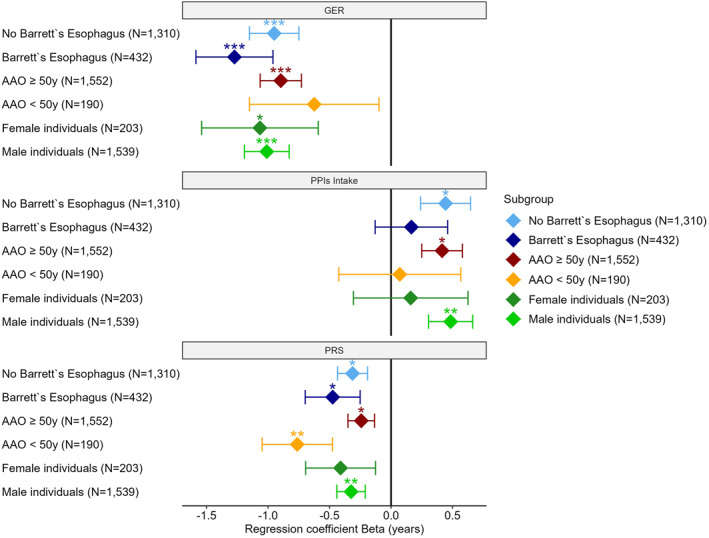
Estimated effects (*β*) of combined use of ASA and PPIs, GER, and PRS on AAO of EAC for patients with BE diagnosis (*N* = 432, dark blue) and without (*N* = 1,310, light blue), male (*N* = 1,539, light green) and female individuals (*N* = 203, dark green), younger (AAO < 50 years, *N* = 190, orange) and older EAC patients (AAO ≥ 50 years, *N* = 1,552, dark red). Each point represents the *β* coefficient from linear regression with 95% confidence interval (CI). A negative *β* indicates earlier AAO, whereas a positive *β* is associated with later AAO. Significance levels: * (*p* < 0.05), ** (*p* < 0.01), and *** (*p* < 0.001). AAO, age at onset; ASA, acetylsalicylic acid; BE, Barrett's esophagus; EAC, esophageal adenocarcinoma; GER, gastroesophageal reflux; PPIs, proton pump inhibitors; PRS, polygenic risk score.

Finally, we tested for sex‐specific effects on EAC AAO, but observed similar trends when female and male patients were analyzed separately (Figure 3). In addition, the presence of a prior BE diagnosis (*N* = 432) did not affect AAO (*β* = 0.27 (± 0.28), *p* = 3.29 × 10^−01^), indicating that AAO was not systematically influenced by BE diagnosis and surveillance. Accordingly, this did not affect all other findings, as similar results were observed in the groups with and without a prior diagnosis of BE (Figure 3). Noteworthy, when focusing on early‐onset EAC (< 50 years, *N* = 190 patients), the PRS showed a markedly stronger association with an earlier AAO (*β* = −0.76 (± 0.28), *p* = 8.55 × 10^−3^). PPIs intake was associated with later AAO in all subgroups. Among patients with a high PRS (PRS > 75%, *N* = 335), the group of non‐smokers and low‐meat consumers showed a significantly later EAC AAO (mean_AAO_ = 64.43, *N* = 122) than individuals who ever smoked and consumed red meat frequently (mean_AAO_ = 56.23, *N* = 60, *p* = 5.545 × 10^−06^).

For non‐dichotomized data, comparable effects were observed for GER, physical activity, fruit and fish consumption, medication and PRS (Supporting Information S1: Table S2 and Figure S2). Red meat consumption and Western diet showed significant effects on earlier EAC AAO. When stratified for current and former smoking, current smoking was significantly associated with an earlier AAO (*β* = −1.08 (± 0.26), *p* = 3.26 × 10^−05^).

## 5 Discussion

This study represents the largest questionnaire‐based analysis to date investigating clinical, dietary, lifestyle, genetic factors, and co‐medication influencing EAC development. Significant effects on earlier AAO among EAC patients were observed for GER, smoking and a higher PRS, while effects on later AAO were significant for physical activity, fish and fruit consumption. Among co‐medication, combined usage of PPIs and acetylsalicylic acid ASA showed the most significant effect on later AAO, whereas PPIs and ASA intake alone had less significant effects, respectively.

Our findings on smoking and GER corroborate the etiological relevance of both exposures on EAC development [2] and underline the quality of our patient phenotypes. However, BMI did not show a significant association with an earlier AAO in our analysis. This points to the challenge of accurately determining BMI at AAO when assessed retrospectively at AAE. Moreover, the BMI was likely confounded by cancer‐related changes in many patients such as dysphagia or weight loss during chemotherapy at advanced EAC stages.

Particularly supporting our findings, physical activity and a vegetable‐rich diet have been suggested to confer EAC protection, although the supporting evidence is limited, while a fruit‐rich diet and alcohol consumption showed no effects on EAC [10]. Because we used dichotomized data based on questionnaires for regression models, the cut‐offs may have been set differently than in other studies on EAC epidemiology.

One of the key findings of our study is that the combined use of PPIs and ASA, both cost‐effective medications, delays EAC development. Although few studies have suggested a protective effect of PPIs and NSAIDs against EAC development due to acid‐suppressant and anti‐inflammatory effects [1, 2, 7], their use for chemoprevention in a broad patient population is not established in clinical practice [10]. Our data confirms the results of these studies in a large patient cohort, even if the initial indication for ASA was likely due to cardiovascular diseases.

We found a significant association between a higher PRS and earlier AAO of EAC, which was particularly pronounced among younger patients (AAO < 50 years). This observation is consistent with other multifactorial diseases and is biologically plausible, as genetic predisposition exerts its greatest effect at younger ages, when other risk factors are less prominent [11].

Several limitations of our study should be acknowledged. First, we included only European patients. Therefore, it remains to be determined whether our findings can be extended to non‐European populations. Second, our sample reflects the known male predominance of EAC [1], which may have introduced a bias toward male‐specific effects. Third, because our analysis focused on AAO, the observed associations may be more indicative of disease progression than of its underlying etiology.

In summary, this study represents the largest questionnaire‐based analysis to date investigating potential factors influencing EAC. Our findings may shape future prevention strategies by confirming the impact of established risk factors (GER, smoking) and supporting the protective role of a Mediterranean diet. Moreover, we found that the combined use of PPIs and ASA, both cost‐effective medications, delays EAC development. Genetic factors also contribute to EAC development, which is particularly pronounced among younger patients. In the future, this finding may facilitate clinical risk stratification, enabling more intensive screening and targeted modification of modifiable risk factors in individuals with a high PRS compared with those with a lower PRS.

## Author Contributions

J.S., and I.G. designed the questionnaires. I.G., N.K., M.Ve., H.A., A.M., C.G., R.T., A.E., C.J.B., H.L., H.M., T.R., T.T.W., M.M., U.W.D., and M.Vi. collected the data. V.K., J.B., D.H., A.M.H., J.R., and M.M.N. processed the data. V.K., and C.M. designed the models. V.K. performed all analyses and designed the figures. V.K., and J.S. wrote the manuscript with input from J.R., R.T., M.V., I.G., A.P.T., and C.M. C.M., and J.S. conceived the study and were in charge of overall direction and planning.

## Funding

The authors have nothing to report.

## Ethics Statement

This study utilized data which involved human participants and was approved by Landesärztekammer Rheinland‐Pfalz 837.095.11 (7637), Faculty of Medicine, University of Leipzig 307/15‐ek, University of Leipzig 204/19‐lk.

## Consent

Participants gave informed consent to participate in the study before taking part.

## Conflicts of Interest

The authors declare no conflicts of interest.

## Supporting information


Supporting Information S1

## Data Availability

The data that support the findings of this study are available from the corresponding author upon reasonable request.
