# Supplementary material for: Clinical, Dietary, Lifestyle and Genetic Factors Associated With Age at Onset of Esophageal Adenocarcinoma
Source: United European Gastroenterol J. 2026 Jun 11;14(5):e70236. doi: 10.1002/ueg2.70236 (PMC13254487; doi:10.1002/ueg2.70236)

**Clinical, dietary, lifestyle and genetic factors associated with age at onset of esophageal adenocarcinoma**

**Supplement: Table of contents**

**Supplementary methods**

| Questionnaire for Barrett’s carcinoma patients | 2 |
| --- | --- |

**Supplementary tables**

| Supplementary Table 1: SNPs used for construction of PRS for Barrett’s esophagus and carcinoma, adapted from Schröder et al. 2023. | 5 |
| --- | --- |
| Supplementary Table 2: Results of linear regression on AAO for 1,742 included records from EAC patients in non-dichotomized data. | 6 |

**Supplementary figures**

| Supplementary Figure 1: Correlation matrix for dichotomized variables of interest (N=19). | 7 |
| --- | --- |
| Supplementary Figure 2: Estimated effects (β) of clinical, demographic, lifestyle, and dietary factors as well as PRS on AAO of EAC in non-dichotomized data. | 8 |

**Supplementary methods**

**Questionnaire for Barrett’s carcinoma patients.**

**I. Personal data**

1. Surname: ______________________________________

2. First name: _____________________________________

3. Sex: Male ❑ Female ❑

4. Date of birth ____ . ____ . _________

5. Height _____ cm

6. Approximate weight at the time of the diagnosis of Barrett’s carcinoma ______ kg (+/-5kg)

**II. Information on education**

7. Education:

- No school leaving certificate ❑
- Secondary modern school ❑
- Graduation from other types of school/vocational training ❑
- University entrance qualification without university degree ❑
- University entrance qualification with university degree ❑

**III. Information on dietary habits**

8. Type of nutrition before the diagnosis of Barrett’s carcinoma:

- Vegan food (exclusively vegetable diet) ❑
- Vegetarian food, including dairy products and/or eggs ❑
- Mixed meals with a large proportion of plant-based foods ❑
- High meat diet (i. e. > 2-3 portions of meat (e.g. schnitzel, steak etc.) per week) ❑

9. How frequently did you eat *red meat* (e.g. beef, pork) prior to the diagnosis of Barrett’s carcinoma?

- Red meat only occasionally (maximum 1x/week) ❑

approx. 3x per week ❑

approx. 5x per week ❑

daily ❑

10. How was the meat most often prepared before the diagnosis of Barrett’s carcinoma?

- Fried or grilled ❑
- Other types of preparation ❑

11. How often did you eat *fish* before the diagnosis of Barrett’s carcinoma?

- never ❑
- less often than 1 x per week ❑
- 1-3 x per week ❑
- more than 3x per week ❑

12. How often did you eat fruit (incl. fruit juices) before the diagnosis of Barrett’s carcinoma?

- maximum 1x per week ❑
- 1-3x per week ❑
- Every day ❑

13. How often did you eat *vegetables* prior to the diagnosis of Barrett’s carcinoma (including salads and vegetable juices)?

- maximum 1x per week ❑
- 1-3x per week ❑
- Every day ❑

**IV. Information on lifestyle**

14. Did you regularly drink alcohol prior to the diagnosis of Barrett’s carcinoma?

- no ❑
- yes ❑

15. Did you smoke prior to the diagnosis of Barrett’s carcinoma?

- no, never ❑
- no longer, for ______ years. During my time as a smoker, on average approximately ______ pieces/ for ______ years
- yes, on average approximately _____ pieces/day, for _____ years

16. Did you regularly exercise or were physically active prior to the diagnosis of Barrett’s carcinoma (multiple answers possible)

- no, I did not do any sports ❑
- yes, I used to do cardiovascular training (e.g. jogging, walking) or endurance training (e.g. football, tennis, etc.) ❑
- yes, I did strength building training ❑

**V. Specific questions on the clinical factors**

17. Are you affected by any internal diseases (e.g. immunological, cardiovascular, high blood pressure, lung diseases)?

- no ❑
- yes ❑

18. Have you been diagnosed with Barrett’s carcinoma?

- no ❑
- yes ❑

19. Have you been diagnosed with Barrett’s esophagus?

- no ❑
- yes ❑

20. Did you suffer from heartburn prior to the diagnosis of Barrett’s carcinoma?

- no, I never had any heartburn ❑
- yes, I had heartburn on average less than once a week ❑
- yes, I had heartburn approximately once a week ❑
- yes, I had heartburn 2-3x per week ❑
- yes, I had heartburn 4-6x per week ❑

21. Have you been diagnosed with Peutz-Jeghers syndrome?

- no ❑
- yes ❑

22. Have you been diagnosed with familial gastric cancer syndrome?

- no ❑
- yes ❑

23. Have you been diagnosed with hereditary nonpolyposis colorectal cancer (HNPCC, Lynch syndrome)?

- no ❑
- yes ❑

24. Have you been diagnosed with familial adenomatous polyposis (FAP)?

- no ❑
- yes ❑

**VI. Medication history**

25. Prior to the diagnosis of Barrett’s carcinoma I have taken medication for heartburn (multiple designations possible)

- no ❑
- yes ❑
  - antacids: daily ❑ 2-3x per week ❑ >1x per week ❑
  - histamine-receptor-antagonists: daily ❑ 2-3x per week ❑ >1x per week ❑
  - Proton pump inhibitors (PPIs): daily ❑ 2-3x per week ❑ >1x per week ❑

26. Other medications I have taken regularly over a period of approximately 2 years prior to the

diagnosis of Barrett’s carcinoma (multiple designations possible):

- ASA (Aspirin) ❑
- COX2-inhibitors (e.g. celecoxib, etoricoxib, parecoxib) ❑
- Ibuprofen ❑
- Diclofenac ❑
- ACE-inhibitors ❑
- Cortisone ❑
- Insulin therapy ❑
- Women only: contraceptives ❑
- Other: _______________________

_______________________

_______________________

**Supplementary tables**

**Supplementary Table 1: SNPs used for construction of PRS for Barrett’s esophagus and carcinoma, adapted from Schröder et al. 2023.**

*Chr – chromosome, EA/OA – effect allele/other allele, β – β-coefficient, SE – standard error, P – p-values. The distance between the lead-associated SNP and the nearest gene is given in brackets in kilobases (kb) next to the gene symbol.*

| **Marker** | **Chr** | **Position** | **EA/OA** | **β** | **SE** | **P** | **Mapping or nearest genes** |
| --- | --- | --- | --- | --- | --- | --- | --- |
| rs9306895 | 2 | 20 878 153 | T/C | −0.122 | 0.015 | 1.90×10^−15^ | *GDF7* (c.*6968T>C) |
| rs12987825 | 2 | 153 229 211 | T/C | 0.090 | 0.016 | 3.88×10^−8^ | *FMNL2* |
| rs146917555 | 2 | 200 024 714 | A/AAATT | −0.099 | 0.015 | 6.66×10^−11^ | *SATB2* (109 kb) |
| rs2687197 | 3 | 70 922 320 | T/C | 0.111 | 0.016 | 3.18×10^−12^ | *FOXP1* (79 kb) |
| rs60849513 | 3 | 157 311 423 | T/G | 0.120 | 0.019 | 9.56×10^−11^ | *SLC66A1L* |
| rs149252763 | 4 | 124 562 153 | G/GGGAAGGAA | 0.137 | 0.019 | 6.45×10^−13^ | *SPRY1* (237 kb) |
| rs144823843 | 5 | 642 293 | A/G | −0.117 | 0.018 | 1.91×10^−10^ | *CEP72* |
| rs77013824 | 5 | 28 954 518 | T/TAAG | 0.094 | 0.015 | 2.95×10^−10^ | *CDH9* (1915 kb) |
| rs72760500 | 5 | 43 785 673 | A/G | 0.227 | 0.031 | 2.42×10^−13^ | *NNT* (78 kb) |
| rs3111642 | 5 | 53 676 856 | A/G | 0.114 | 0.020 | 1.16×10^−8^ | *ARL15* (70 kb) |
| rs9463175 | 6 | 9 510 030 | T/C | 0.093 | 0.016 | 2.99×10^−9^ | *TFAP2A* (886 kb) |
| rs622217 | 6 | 160 766 770 | T/C | 0.130 | 0.015 | 3.28×10^−18^ | *SLC22A3* (2 kb) |
| rs2188554 | 7 | 117 040 117 | A/G | 0.128 | 0.019 | 3.12×10^−11^ | *ASZ1* |
| rs111613945 | 8 | 11 434 415 | CTCGGTTT/C | −0.096 | 0.015 | 9.79×10^−11^ | *BLK* (12 kb) |
| rs7045553 | 9 | 100 307 789 | T/C | −0.098 | 0.015 | 1.69×10^−10^ | *TMOD1* |
| rs1247942 | 12 | 114 673 723 | C/G | −0.089 | 0.015 | 4.48×10^−9^ | *TBX5* (118 kb) |
| rs848092 | 14 | 37 268 797 | A/G | 0.086 | 0.016 | 3.41×10^−8^ | *SLC25A21* |
| rs4646614 | 15 | 58 264 792 | A/ATAGT | −0.100 | 0.015 | 2.68×10^−11^ | *ALDH1A2* |
| rs35631104 | 15 | 97 557 787 | G/GA | 0.126 | 0.017 | 1.18×10^−13^ | *NR2F2* (674 kb) |
| rs3950627 | 16 | 86 436 343 | A/C | −0.099 | 0.015 | 3.39×10^−11^ | *FOXF1* (107 kb) |
| rs7187365 | 16 | 86 511 915 | T/C | 0.116 | 0.019 | 1.19×10^−9^ | *FOXF1* (32 kb) |
| rs199620551 | 19 | 18 804 294 | T/TG | −0.094 | 0.015 | 3.43×10^−10^ | *CRTC1* |
| rs6104657 | 20 | 10 738 950 | A/C | 0.109 | 0.019 | 1.52×10^−8^ | *JAG1* (84 kb) |

**Supplementary Table 2:** **Results of linear regression on AAO for 1,742 included records from EAC patients in non-dichotomized data.**

*Beta – β-coefficient, SE – standard error, P – false discovery rate (FDR) corrected p-values, Group – category of analyzed lifestyle factors or clinical information, AAO – age at onset, ASA – acetylsalicylic acid, BMI – body mass index, GER – gastroesophageal reflux, PPIs – proton pump inhibitors, PRS – Polygenic Risk Score, PY: pack years. Significance levels were defined as follows * (P<0.05), ** (P<0.01), and *** (P<0.001). Variables are sorted in ascending order by beta value.*

| **Variable** | **Beta** | **SE** | **P** | **Group** |
| --- | --- | --- | --- | --- |
| **Current vs Former Smoking** | -1.079 | 0.259 | 3.26x10-05 *** | Lifestyle Factors |
| **Western Diet** | -0.406 | 0.181 | 2.50x10-02 * | Dietary Factors |
| **GER** | -0.380 | 0.062 | 1.28x10-09 *** | Clinical Factors |
| **PRS** | -0.313 | 0.117 | 7.59x10-03 ** | Polygenic Risk Score |
| **Red Meat Consumption** | -0.301 | 0.112 | 7.22x10-03 ** | Dietary Factors |
| **Alcohol Consumption** | -0.246 | 0.174 | 1.56x10-01 | Lifestyle Factors |
| **BMI** | -0.008 | 0.017 | 6.36x10-01 | Clinical Factors |
| **Ever Smoking** | 0.005 | 0.198 | 9.80x10-01 | Lifestyle Factors |
| **Lifelong PY Dosage** | 0.008 | 0.005 | 1.25x10-01 | Lifestyle Factors |
| **High Education Level** | 0.030 | 0.071 | 6.69x10-01 | Demographic Factors |
| **Frequent PPIs Intake** | 0.118 | 0.064 | 6.65x10-02 | Medication |
| **PPIs w/o ASA** | 0.170 | 0.188 | 3.66x10-01 | Medication |
| **Vegetables Consumption** | 0.172 | 0.136 | 2.09x10-01 | Dietary Factors |
| **Fruits Consumption** | 0.268 | 0.119 | 2.40x10-02 * | Dietary Factors |
| **Fish Consumption** | 0.341 | 0.150 | 2.27x10-02 * | Dietary Factors |
| **ASA w/o PPIs** | 0.346 | 0.296 | 2.44x10-01 | Medication |
| **Barrett`s Esophagus** | 0.407 | 0.198 | 3.95x10-02 * | Clinical Factors |
| **PPIs Intake** | 0.436 | 0.178 | 1.43x10-02 * | Medication |
| **Physical Activity** | 0.496 | 0.174 | 4.51x10-03 ** | Lifestyle Factors |
| **ASA Intake** | 0.715 | 0.235 | 2.39x10-03 ** | Medication |
| **ASA with PPIs** | 1.046 | 0.341 | 2.20x10-03 ** | Medication |

**Supplementary figures**

**Supplementary Figure 1:** **Correlation matrix for dichotomized variables of interest (N=19).**

The Pearson correlation coefficient (r) is represented in this heatmap by red for negative r, and by blue for positive r. r<|0.4| was considered as weak correlation, while r>|0.6| indicates strong correlation between two variables. A strong correlation is evident between AAE and AAO, as well as between medication intake and combined intake.

*AAE: age at examination, AAO: age at onset, ASA: acetylsalicylic acid, BE: Barrett’s esophagus, BMI: body mass index, GER: gastroesophageal reflux, PPIs: proton pump inhibitors, PRS: Polygenic Risk Score.*

**
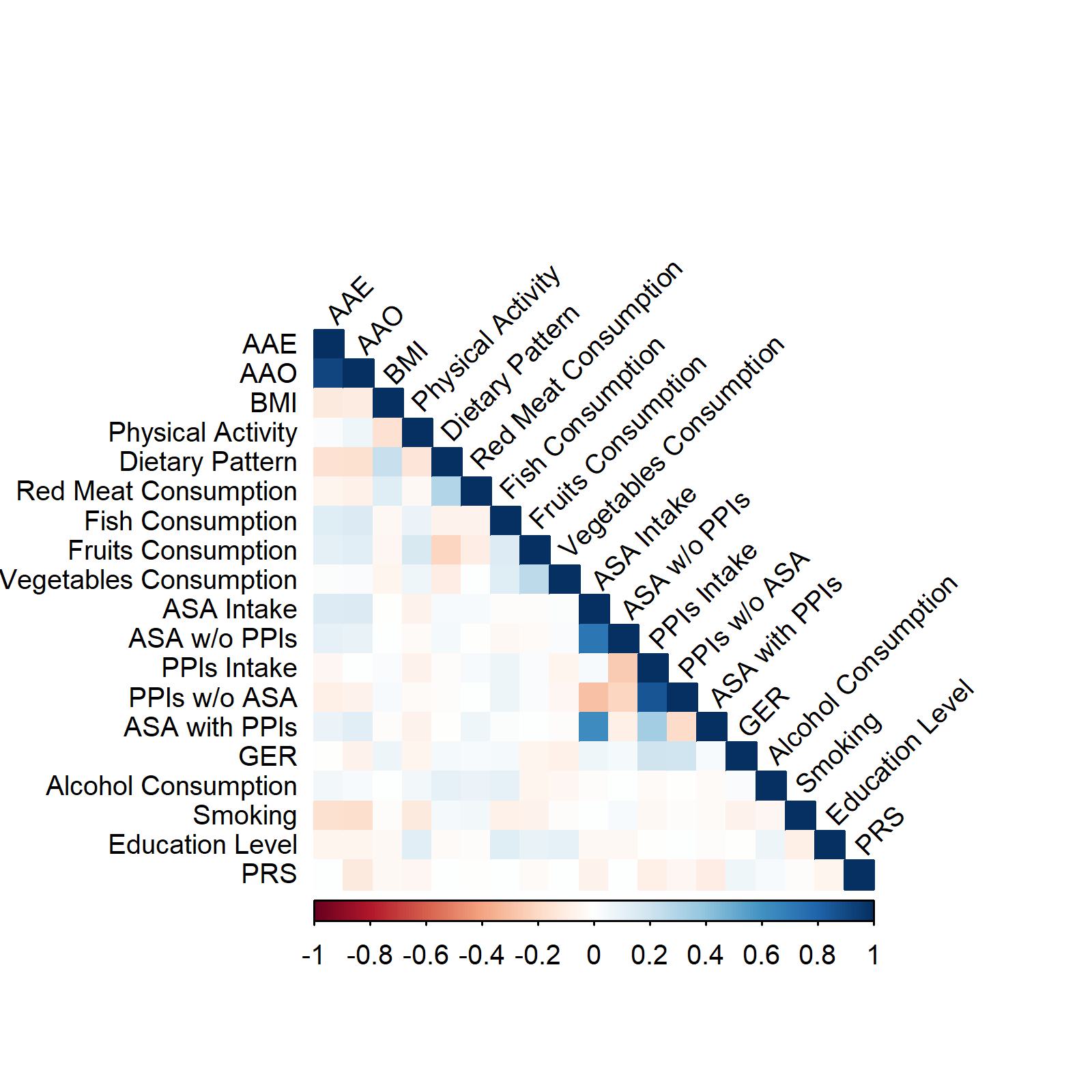
**

**Supplementary Figure 2:** **Estimated effects (β) of clinical, demographic, lifestyle, and dietary factors as well as PRS on AAO of EAC in non-dichotomized data.**

Each point represents the β coefficient from linear regression with 95% confidence interval (CI). A negative β indicates earlier AAO, while a positive β is associated with later AAO. Significance levels: * (P<0.05), ** (P<0.01), and *** (P<0.001).

*AAO: age at onset, ASA: acetylsalicylic acid, BE: Barrett’s esophagus, BMI: body mass index, EAC: esophageal adenocarcinoma, GER: gastroesophageal reflux, PPIs: proton pump inhibitors, PRS: Polygenic Risk Score, PY: pack years.*


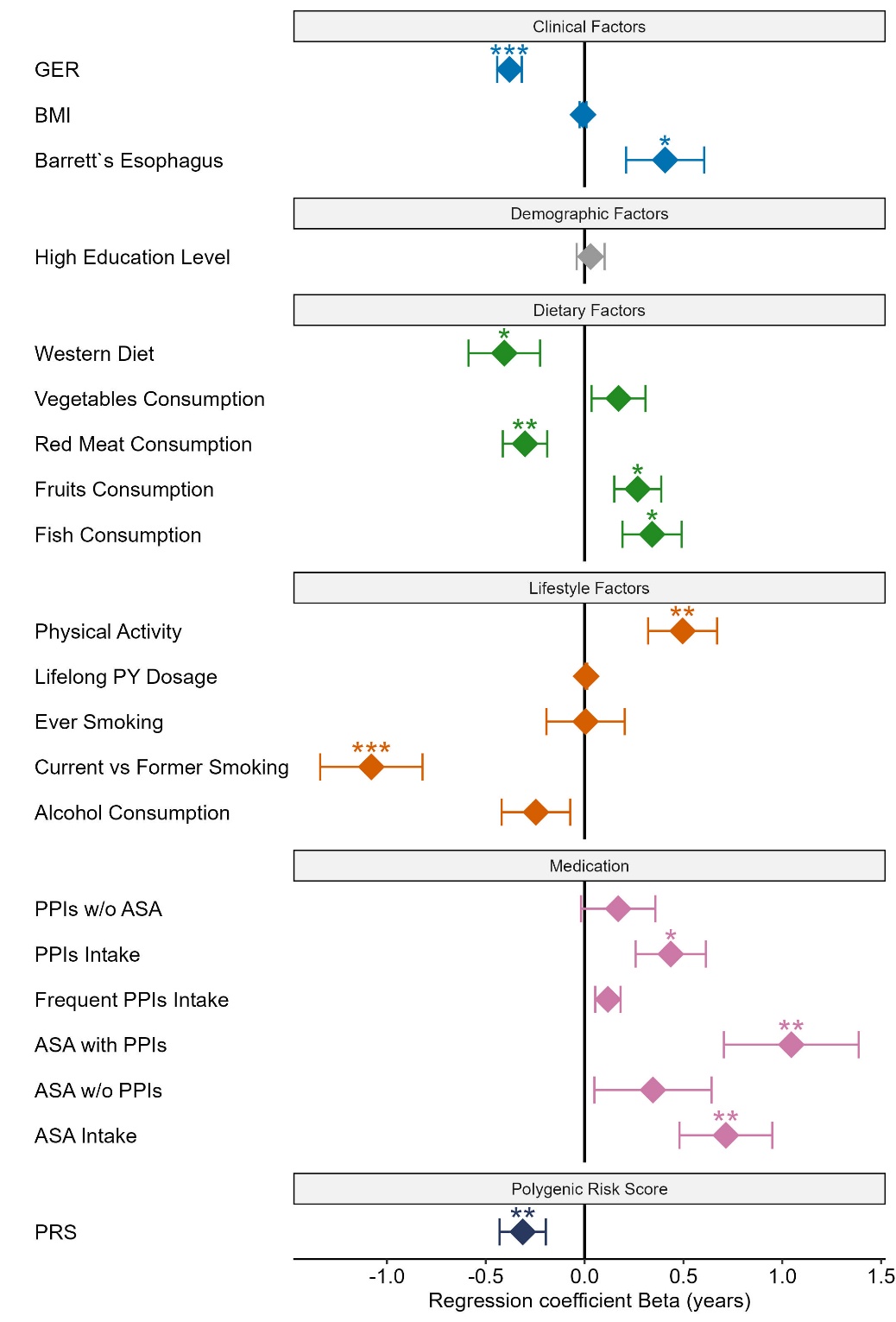

Supplement: Supplementary file 1 — Supporting Information S1 [file UEG2-14-e70236-s001.docx]
